# MicroRNAs in Coronary Heart Disease: Ready to Enter the Clinical Arena?

**DOI:** 10.1155/2016/2150763

**Published:** 2016-11-02

**Authors:** Elena Cavarretta, Giacomo Frati

**Affiliations:** ^1^Department of Medical-Surgical Sciences and Biotechnologies, Sapienza University of Rome, Corso della Repubblica 79, 04100 Latina, Italy; ^2^Department of AngioCardioNeurology, IRCCS NeuroMed, 86077 Pozzilli, Italy

## Abstract

Coronary artery disease (CAD) and its complication remain the leading cause of mortality in industrialized countries despite great advances in terms of diagnosis, prognosis, and treatment options. MicroRNAs (miRNAs), small noncoding RNAs, act as posttranscriptional gene expression modulators and have been implicated as key regulators in several physiological and pathological processes linked to CAD. Circulating miRNAs have been evaluated as promising novel biomarkers of CAD, acute coronary syndromes, and acute myocardial infarction, with prognostic implications. Several challenges related to technical aspects, miRNAs normalization, drugs interaction, and quality reporting of statistical multivariable analysis of the miRNAs observational studies remain unresolved. MicroRNA-based therapies in cardiovascular diseases are not ready yet for human trials but definitely appealing. Through this review we will provide clinicians with a concise overview of the pros and cons of microRNAs.

## 1. Introduction

Approximately every 42 seconds, a US American will suffer a heart attack. Cardiovascular and cerebrovascular diseases represent the leading cause of death worldwide, even if death rates have fallen from 1968 to the present [1]. Moreover, the lifetime risk for coronary heart disease varies drastically as a function of risk factor profile. With an optimal risk factor profile, lifetime risk for CHD is 3.6% for men and <1% for women; with ≥2 major risk factors, it is 37.5% for men and 18.3% for women [2]. Therefore, a correct identification of those individuals by specific biomarkers related to diagnosis, screening, staging, monitoring, surveillance, prognosis, and treatment selection would be of pivotal importance. Genetics, intermediate phenotype, life-style, and other environmental triggers are directly involved in the pathogenesis of coronary artery disease (CAD). The estimated heritability of CAD ranges from 30 to 60% [3, 4]. Recently, several studies highlighted that the genetics of CAD largely derives from the cumulative effect of multiple common risk alleles, emphasizing the individual but cumulative small effect size rather than rare variants with large effects on CAD risk. Despite this finding, there has been less success in understanding the function of the novel loci; in fact the majority of these loci are in noncoding regions of the genome [5]. Even if most of our genome does not encode for proteins and it is extensively transcribed anyway, generating non(protein) coding RNAs. Short noncoding RNAs of approximately 22–24 nucleotides, microRNAs, are widely recognized posttranscriptional gene regulator, while longer (>200 nucleotides) noncoding RNAs are now also recognized to play important roles in gene regulation and function [6]. MicroRNAs in cardiovascular disease are gaining momentum as possible novel biomarkers in the diagnosis and prognosis of coronary artery disease, acute coronary syndrome, and heart failure. Nowadays, diagnosis of acute coronary syndrome relies on symptoms, electrocardiogram abnormalities, and troponin quantification, with much interest in developing new rule-out and rule-in strategies or possible new promising biomarkers. In the literature, there is much ado about a possible clinical role of microRNAs in coronary heart disease. We aimed to review the pros and cons of microRNAs use in coronary heart disease applied to the clinical setting.

## 2. MicroRNAs

### 2.1. MicroRNA Biology

In 1993, Lee et al. [7] discovered that the* C. elegans* gene* lin-4* (a gene controlling the nematode larval development) did not encode for a protein but for small noncoding RNAs. A longer one (61 nucleotides) was then cleaved and folded in a stem-loop of 22 nucleotides. This* lin-4* derived RNAs had antisense complementarity to multiple sites in the 3′-UTR of the* lin-14* gene, with a final result of reducing the amount of LIN-14 protein, without changing the amount of* lin-14* messenger RNA (mRNA) [8]. This short lin-4 RNA is the founding member of the microRNAs family. The second member of this family, let-7, had to wait until the year 2000 to be described by Reinhart et al. [9]. Since then the miRNAs family has markedly expanded and more than 2000 different miRNAs sequences have been described and catalogued in miRBase [10]. MicroRNAs function as gene regulators acting on mRNAs translation, with inhibition of protein synthesis. Basically, different miRNAs may target a given mRNA in different binding sites (convergent microRNA pathway) or a single miRNA may target multiple different mRNAs (divergent miRNA pathway) [11]. There are specific types of software to predict which mRNAs may be the target of a specific miRNA (TargetScan, http://www.targetscan.org/; miRanda, http://www.microRNA.org/; TarBase (http://www.microrna.gr/tarbase).

**Figure 1:**
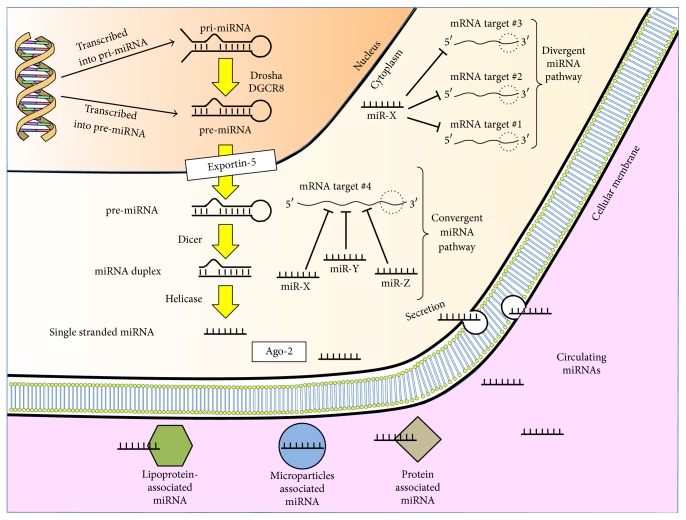
MicroRNAs biogenesis and function. In the nucleus, DNA is transcribed into pri-miRNA and then cleaved by Drosha to produce pre-miRNA in the canonical microRNA biogenesis pathway. Noncanonical biogenesis pathways exist. Pre-miRNA is then moved in the cytoplasm by Exportin-5 where another RNase III, Dicer cleaves it into a microRNA duplex, to finally obtain a single stranded microRNA. The microRNA can exercise his action internally or in a cell-to-cell interaction, through convergent or divergent microRNA pathways. Circulating microRNAs are usually associated with lipoprotein, protein, exosome, and microvesicles. See text for further details. Ago-2: Argonaute protein 2; miRNA: microRNA; mRNA: messenger RNA.

MicroRNAs biogenesis is resumed in Figure 1. Briefly, in the canonical miRNAs biogenesis pathway, primary miRNAs (pri-miRNAs) of hundreds or thousands of nucleotides are synthesized from DNA by the enzyme RNA polymerase II in the nucleus. Pri-miRNAs, folded in the hairpin structure, are then cleaved by the ribonuclease III Drosha with the cofactor DGCR8, to form the microprocessor complex, producing the preliminary miRNAs (pre-miRNAs) of 70–100 nucleotides. The pre-miRNAs are transported into the cytoplasm by Exportin-5 where another ribonuclease III, Dicer, and its cofactor TRBP, cleave them into the shorter, double stranded immature microRNAs. The miRNA-miRNA^*∗*^ duplex is then transferred to the Argonaute protein family (Ago) that undergoes conformational changes to allow binding of the miRNAs duplex. In the strand selection process, the passenger strand or miR^*∗*^ is discarded while the leading strand or miR is incorporated into the RISC (RNA-Induced Silencing Complex). Into the RISC, the miRNA presents the seed sequence at an interface where it can interact with a region of the mRNA within its 3′-UTR [12]. Other noncanonical miRNAs biogenesis pathways have been described [13].

### 2.2. Circulating MicroRNA

MicroRNAs can act intracellularly or can be actively secreted by cells and contribute to intercellular or cell-tissue communication [14]. Circulating microRNAs are stable despite the high extracellular RNase activity, due to their packaging in apoptotic bodies, microvesicles, and exosomes or association with lipoprotein, protein as the Argonaute family and other RNA binding proteins. Microvesicles and exosomes are fundamentally different, the first being smaller and heterogeneous in size, ranging from 100nm to 1*μ*m, derived from the plasma membrane and released by budding and fission of the membrane, while the latter being formed intracellularly via endocytic invagination and then released into a multivesicular body [15]. Since the discovery in 2008 of miRNAs in blood [16], circulating miRNAs have been found in blood, urine, breast milk [17], saliva [18], tears, and other body fluids [19]; their potential use as serum biomarkers has become more appealing. Biomarkers should be divided into two different categories, depending on their possibility to change over time: genetic markers, stable over time, and dynamic markers, which may change mainly over time. A biomarker should be noninvasively obtained and have a high degree of sensitivity and specificity, permitting early diagnosis of disease. Moreover, a biomarker should have time-related changes in the disease course, a long half-life within the sample, allowing rapid and cost-effective laboratory detection. Some of these essential characteristics are shared by circulating miRNAs: their small size, a simple chemical composition, their high stability in boiling water, their resistance to extreme pH changes, prolonged room temperature stays or repeated freeze-thawing [19, 20], less complexity in comparison with proteins, and a cost-effective quantification by real-time polymerase chain reaction (qRT-PCR).

## 3. MicroRNAs in Coronary Heart Disease

### 3.1. MicroRNAs in Acute Coronary Syndrome

**Figure 2:**
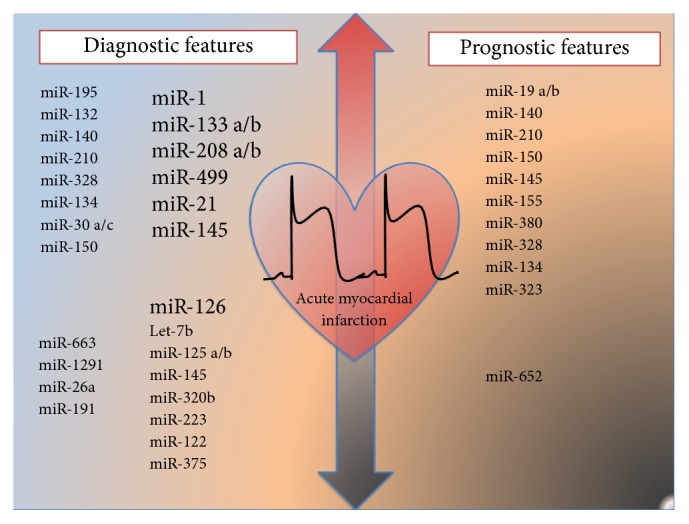
Circulating microRNAs associated with diagnostic and prognostic features in acute coronary syndrome (ACS). microRNAs in bigger font have been associated with ACS in more than one study. See Table 1 for further details.

In 2010, five authors [21–25] independently reported a possible role for cardiomyocyte-enriched miRNAs in the diagnosis of acute myocardial infarction (AMI). Specifically, in these studies taken together, miR-1, miR-133a, miR-133b, miR-208, and miR-499 were found upregulated in plasma of AMI patients.  Figure 2 resumes the potential miRNAs up- and downregulated in AMI with diagnostic and prognostic implications. More than 30 studies analyzed the possible diagnostic microRNAs signature in AMI and other possible miRNA candidates have been proposed, but further validation studies are needed [26]. Years later, almost the same miRNAs are recognized as cardiac-enriched and proposed as possible biomarkers among several other miRNAs by 2 different authors. In 2014, a meta-analysis [27] of 19 studies evaluated the specificity and sensitivity of miR-1, miR-133a, miR-208b, and miR-499 in AMI. Cheng et al. concluded that miR-499 and miR-133a are possible biomarkers of AMI, showing a sensitivity of 0.88 (95% CI: 0.86–0.90; *p* = 0.0000); a specificity of 0.87 (95% CI: 0.84–0.90; *p* = 0.0000) and a sensitivity of 0.89 (95% CI: 0.83–0.94; *p* = 0.0047); a specificity of 0.87 (95% CI: 0.79–0.92; *p* = 0.0262), respectively. More recently, in a systematic review [28] the authors proposed that only cardiomyocyte-enriched miRNAS, miR-1, miR-133a/b, miR-145, miR-208a/b, and miR-499(a) in plasma and/or serum are potential biomarkers for the diagnosis of coronary heart disease.

Devaux et al. [29] presented the largest multicenter study on miRNAs in 1155 unselected patients with acute chest pain. miR-208b provided the highest diagnostic accuracy in AMI but still this was lower than that of the fourth-generation or high-sensitivity cardiac troponin T (cTnT). None of the six miRNAs provided added diagnostic value when combined with cTnT.

The prognostic role of miRNAs is encouraging. Few studies [29–34] have evaluated the role of miRNAs as prognostic biomarkers with controversial results; see Table 1 for further details. Very recently Karakas et al. [34] found for the first time that peripheral-blood miRNAs (miR-132, miR-140-3p, and miR-210) could predict CV mortality in a large cohort of ACS and stable CAD patients, while none of the cardiomyocyte-enriched miRNAs evaluated by Devaux et al. [29] predicted long-term mortality at 2-year follow-up, neither miR-208b nor miR-499 were significant predictors of mortality [33, 35]. Widera et al. [31, 34] found that miR-133a and miR- 208b levels were significantly associated with the risk of death in ACS patients, but in adjusted analysis their independent association with outcome was lost. Matsumoto et al. [32, 36] proposed 2 different sets of miRNAs with prognostic implications at 1-year follow-up post-AMI, but validation studies are needed for both.

### 3.2. Controversies in the Role of miRNAs as Biomarkers of AMI

The role of miRNAs has novel biomarkers in the early diagnosis of AMI is debated. The index test is high-sensitivity cardiac troponin, which is widely used in clinical practice and shows high accuracy in AMI diagnosis; therefore, it is very difficult for new biomarkers to demonstrate significant added value on top of cardiac troponins. Moreover, the 3rd universal definition of AMI relies on symptoms and detection of troponin-positive myocardial necrosis, even if the unspecific elevation of troponin levels can be present in case of nonischemic heart failure (HF), renal failure, myocarditis, arrhythmias, and pulmonary embolism due to myocardial injury [37]. Wang et al. [25] reported that, in AMI patients, miR-208 levels were not altered by chronic kidney disease or trauma, as it happens for troponins. Actually miRNAs can reduce this gap and provide additional accuracy in the diagnosis of AMI, as some miRNAs became detectable when initial troponin was still negative or within 3h of symptom onset [38].* De iure* miRNAs become detectable earlier than high-sensitivity troponin, theoretically allowing a faster rule-in/rule-out of chest pain patients; one of the major limits of cTnT is that multiple dosage at different time is needed and patients are ordered to stay in the emergency room for 3–6 h after arrival.* De facto* measurement of circulating miRNAs requires qRT-PCR, which is a time-consuming technique, in comparison with detection of hs-cTnT (approximately 30 min) and the 2015 ESC guidelines recommend the use of a rapid rule-out protocol (0 h and 1 h or 0 and 3 h) when hs-cTnT is available [39]. The use of qRT-PCR is currently the limiting factor in terms of rapid detection of circulating miRNAs. In the future, the availability of newer, faster, and cost-effective techniques may overcome this limit.

### 3.3. MicroRNAs in Coronary Artery Disease

The ability to distinguish stable from unstable angina pectoris patients would be a great advance in CAD management, but this promise is far from being fulfilled, as concluded by D'Alessandra et al. [40]. Several miRNAs, as cardiomyocyte-enriched (miR-133, miR-208a) [41], endothelial cell-enriched (miR-126, miR-17-92a cluster), vascular smooth cell (miR-143/145) and inflammatory cell-enriched (miR-155), and platelet-enriched (miR-199a) miRNAs, were associated with CAD, while lipometabolism-related miR-122 and miR-370 increased as the severity of CAD quantified by the Gensini score increased [42]. Previously, miR-126 has been proposed as a prognostic marker of incident myocardial infarction in the general population [43], result partially confirmed by Jansen et al. [44] who reported that only microvesicles-associated miR-126 and miR-199a predict the occurrence of CV events in patients with stable CAD. A more comprehensive review has been recently published [45].

## 4. Technical Aspects of miRNAs Quantification

The sensible differences and heterogeneous results reported in ACS and CAD studies can be partly explained by some technical aspects and drugs interaction. Quantification of miRNAs transcripts by qRT-PCR implicates data normalization with endogenous and exogenous reference genes for data correction. Data from qRT-PCR can be analyzed using absolute or relative quantification. Absolute quantification defines expression levels in absolute numbers of copies by relaying the PCR signal to a standard curve. Relative quantification determines fold changes in expression between two samples, normalizing the gene of interest for a housekeeping gene in the same sample to obtain a fold change. One of the most frequently used normalizers is the small noncoding RNA RNU6, which is not a miRNA and could not perfectly reflect the miRNAs biochemical characteristics. miR-16 is another frequently used normalizer because it is highly expressed and relatively invariant. The choice of the reference gene can be challenging as an optimal normalization strategy is missing. Consequently, the choice of which miRNAs should be used as internal controls for circulating miRNAs assessment could lead to ambiguous data interpretation, misleading conclusions, and erroneous biological predicted effect, impairing comparison between studies and meta-analysis of data. The use of more than 1 reference gene increases the accuracy of quantification; for example, the combined use of miR-16 and other miRNAs could reduce the potential bias compared to the use of a single reference gene [46]. Some authors stated that, in the lack of a shared housekeeping miRNA, miRNAs expressions do not require an internal control and could be normalized to serum volume [47]. However, this strategy has been demonstrated to increase the risk of bias and should be avoided. In addition, while searching for the ideal normalization gene candidate, it is pivotal to apply standardization across laboratories for sample preservation, storage, and stability.

Another potentially confounding factor is drug administration. Statins [42], anticoagulation [48], and antiplatelet drugs [49] can affect quantification of miRNAs in blood samples and must be taken into account when assessing circulating miRNAs [50]. To overcome the potential confounding effect of heparin, Kaudewitz et al. suggested normalizing with exogenous* C. elegans* spike-in control [51]. Other options to treat plasma from patients subjected to heparin treatment include digestion with heparinase on purified RNA rather than plasma, optimization of the starting plasma volume, and enrichment of miRNA on silica [52, 53]. To successfully translate miRNA signature in clinical practice it is mandatory to develop and apply a standardization of the operative procedures related to circulating miRNAs analysis. Standardization needs to be applied at several stages, from blood withdrawal to plasma/serum centrifugation, to sample collection and banking, and to RNA extraction and microRNAs quantification, in order to dramatically reduce interlaboratory differences that could generate huge incoherencies in miRNAs analyses. Consequently, bias in the selection, extraction, and quantification of miRNAs generating unexplained contrasting results is widespread in most studies and represent a major limitation to perform a meta-analysis.

## 5. Quality Reporting in Circulating MicroRNAs Observational Studies in Coronary Heart Disease

Several authors reported that the biggest limitation for use of miRNAs as biomarkers is the small sample size of published studies [48, 54]. Not only small sample size, but also the quality of reporting of observational studies is a major issue, due to the lack of randomized double blind trials. Controlling for already mentioned confounders is a crucial step in microRNAs observational studies, to avoid misleading conclusion. To overcome this problem, the use of multivariable models as statistical adjustment techniques is widely encouraged and the validation of assumption of the multivariable regression models should be clearly stated in the methodology. To our knowledge, a quality report on statistical methodology in circulating miRNAs studies has not yet been performed. We reviewed the quality of statistical reporting of 56 studies (see Supplementary Figure S1 and Table S1 for included studies in Supplementary Material available online at http://dx.doi.org/10.1155/2016/2150763) on circulating miRNAs in coronary heart disease (ACS, AMI, and CAD). A list of the Real et al. items reviewed in full-text studies is presented in Table 2, based on Real et al. methods [55]. See Supplementary Materials for complete methods and statistical analysis. Results are resumed in Table 3. The large majority of studies are in fact cohort studies of small sample size (median size 115 patients). Of note, significant differences exist between small (<100 patients) and large sample size (>500 patients) studies in terms of quality reporting of multivariable regression models. A multiple regression was run to predict a quality score >2 from adopted model, journal impact factor, citation/year, and sample size. Among these variables, only sample size statistically significantly predicted quality score, *F*(5, 50) = 22.201, *p* < 0.0001, *R* ^2^ = .689. The totality of larger sample size studies scored at least 3 over 5 checked items, demonstrating a solid methodology and control for confounders. In fact, some miRNAs lost statistical significance when adjusted for confounders [29, 34, 43]. It is highly possible that among the authors of large sample size study a methodologist is included. Small sample size studies without adjustment for confounders of the results contribute to increase heterogeneity and introduce possible bias in the literature. No significant differences exist in terms of article citations per year; highly cited articles can have a robust or a weak methodology. Obviously the first reports were small sample size studies but great breakthroughs in microRNAs biology and function were therefore highly cited. Studies with weak methodology can present contrasting results and then be cited in contrast to more robust studies, creating confusion. Nevertheless, the journal impact factor has definitely a role in assessing the methodological quality of the study and even if it does not reach the full significance in our results, the trend is in favor of a positive correlation between impact factor and high methodological score.

## 6. MicroRNA-Based Therapeutics

Up-to-date microRNAs-based therapies are in their infancy, thus experimental and animal studies are in favor of a potential role in the treatment of CV diseases. In nonhuman primates, inhibition of miR-33a and miR-33b by an anti-miRNA oligonucleotide increased hepatic expression of ABCA1, a key regulator of high density lipoprotein (HDL) biogenesis, and induced a sustained increase in plasma HDL levels over 12 weeks, with reduction of very low density lipoprotein (VLDL) levels [56]. Another study assessed the role of locked nucleic acid-modified antisense miR-92a (LNA-92a) in a model of ischemia/reperfusion injury in pigs and revealed cell-protective, proangiogenic, and anti-inflammatory effects of LNA-92a with reduction of infarct size and improved recovery of cardiac function [57]. Unfortunately these promising results have not yet progressed to human trials. After the seminal studies on miR-21 by Thum [58], a key target in CV diseases would be reduction/inhibition of myocardial fibrosis associated with postischemic cardiopathy, drug-induced or primitive cardiomyopathies [59], but the question is far from being resolved yet [60, 61]. In other fields of medicine miRNA-based therapies are a step forward. In patients with chronic hepatitis C, subcutaneous administration of an antisense oligonucleotide for miR-122 led to successful results with negligible side effects in phases 1 and 2a trial [62] and at long-term follow-up [63].

## 7. Conclusion

Translational research represents a stem of scientific research that helps to make findings from basic science useful for practical applications that enhance human health and well-being. Deeply established on multidisciplinary collaboration, translational research has the enormous potential to move applied science forward. Accordingly, in the cardiovascular system, miRNAs fine-tune complex molecular signaling networks by acting on key target proteins involved in a variety of pathways and cellular processes. Therefore, in the past decade, several studies emphasized the importance of miRNAs as diagnostic and prognostic role in cardiovascular disease and the road travelled so far seems promising for a specific role in coronary heart disease. At present, circulating miRNAs have not entered yet the clinical arena, due to contrasting results, possible confounding factors, presence of small or moderately sized studies of different methodology, sometimes challenging each other, technological requirements, and unstandardized normalization. This complex scenario, in which bordering results contradict themselves, may push researchers, clinicians, and also patients in different directions providing dissimilar effect estimates with mixed results and with benefits ranging from absent to transient and, at most, marginal. In the future, the role of long noncoding RNAs may add novel insight into the posttranscriptional regulation changing the way with which investigators identify novel signal transduction pathways and functional cross-talks developing new therapeutic strategies and micro-RNA based therapies might make the way for human trials with important therapeutic implications. Clinicians must be aware of the pros and cons of microRNAs advent and read critically the fore coming literature.

## Supplementary Material

In the supplementary material there is the search strategy and selection criteria of the articles included in the quality reporting, Abstraction, Quality Appraisal, Effect Estimate, and Citation Count and statistical analysis methodology. Figure S1 is a schematic drawing of the selection of the included studies, based on PRISMA guidelines. In Table S1there are the full references of the studies included in the quality reporting analysis.

## Figures and Tables

**Table 1:** Selected studies on circulating microRNAs with prognostic implications after acute myocardial infarction. ACS: acute coronary syndrome; AMI: acute myocardial infarction; CAD: coronary artery disease; CV: cardiovascular; HF: heart failure; hsTnT: high-sensitivity troponin T; MACE: major adverse cardiac event.

Dysregulated miRNAs	Prognosis	Specimen	Normalization	Study population	Follow-up	References
↑ miR-132, miR-140-3p, miR-210	Predicted CV in ACS patients	Serum	*C. elegans* miR-39	430 ACS patients + 682 stable CAD patients	4 years	Karakas et al. 2016 [34]
↑ miR-208b	Predicted 30-day mortality with moderate accuracy	Plasma	*C. elegans* miRs	1155 chest pain patients	2 years	Devaux et al. 2015 [29]
↑ miR-145 on day 5	Predictive of MACE and CV death within 1 year after AMI	Serum	miR-16	246 STEMI patients	1 year	Dong et al. 2015 [64]
↑ miR- 208b, miR-499	Nonsignificant predictors	Plasma	*C. elegans* miRs	510 AMI patients (113 NSTEMI, 397 STEMI)	2–6 years	Goretti et al. 2013 [35]
↑ miR-155, miR-380	Predictive for cardiac death 1 year after AMI	Serum	—	26 patients who died of CV death within 1 year after AMI + 28 event-free AMI patients	1 year	Matsumoto et al. 2012 [32]
↑ miR-133a, miR-208b	miR-133a and miR- 208b levels were significantly associated with the risk of death and lost their independent association with outcome upon further adjustment for hsTnT	Plasma	*C*. *elegans *cel-miR-54	444 ACS patients	6 months	Widera et al. 2011 [31]
↑ miR-208b, miR-499-5p	Equal to TnT for prognostic 30-day death after AMI	Plasma	miR-17	424 ACS patients	30 days	Gidlöf et al. 2013 [33]
↓ miR-652	Predictive of readmission to the hospital for heart failure within 5 years	Plasma	Synthetic UniSp4 and cDNA synthesis UniSp6	235 ACS patients + 116 healthy controls	5 years	Pilbrowa et al. 2014 [65]
↑ miR-133a	Associated with decreased myocardial salvage, larger infarcts, and more pronounced reperfusion injury but failed to prevent events	Serum	*C. elegans* miR-39	216 STEMI patients	6 months	Eitel et al. 2012 [66]
↑ miR-192, miR-194, miR-34a	Elevated by the early days after AMI in patients who experienced HF	Serum	U6 snRNA	21 AMI patients who developed HF within 1 year after AMI + 65 event-free AMI controls	1 year	Matsumoto et al. 2013 [36]

**Table 2:** Items reviewed on observational studies assessing the value of microRNAs as potential biomarkers for coronary artery disease and acute coronary syndrome. Table adapted by authors from [55].

Item	Issue	Question
(1)	Model assumption and goodness-of-fit	How far away from the data is the selected model?
(2)	Interaction analysis	Is there any potential variable that can modify the estimated effect?
(3)	Sensitivity analysis	Are the findings sufficiently robust, considering the process used to obtain them?
(4)	Crude and adjusted effect estimate	How much does the studied effect change when other variables are taken into account?
(5)	More than one adjusted model specified	Does the estimated effect differ between the different adjusted models, settings, specifications, and so forth?

**Table 3:** Frequency of application of multivariable regression models, based on study features, of the observational studies assessing the value of microRNAs as potential biomarkers for coronary artery disease and acute coronary syndrome.

Variable	Median (1st quartile; 3rd quartile)	Category	*N*	Model assumption	Interaction analysis	Sensitivity analysis	Crude and adjusted effect estimate	More than one adjusted model specified	Reporting at least > 2 items
Articles	—		56	29 (52%)	32 (57%)	30 (54%)	14 (25%)	19 (34%)	34 (61%)
		95% CI		39–64%	44–69%	41–66%	15–38%	23–47%	48–72%

Publication year	2013 (2012; 2014)			*p* = 0.418	*p* = 0.941	*p* = 0.596	*p* = 0.139	*p* = 0.869	*p* = 0.806
		2010-2011	12	7 (58%)	6 (50%)	7 (58%)	0 (0%)	3 (25%)	3 (25%)
		2012-2013	17	7 (41%)	10 (59%)	11 (65%)	5 (29%)	6 (35%)	6 (35%)
		2014-2015	25	13 (52%)	15 (60%)	11 (44%)	8 (32%)	9 (36%)	10 (40%)
		2016	2	2 (100%)	1 (50%)	1 (50%)	1 (50%)	1 (50%)	1 (50%)

Sample size	115 (58; 312)			*p* = 0.129	*p* = 0.138	*p* = 0.054	**p** < 0.001	**p** < 0.001	**p** < 0.001
		<100	30	13 (43%)	14 (47%)	12 (40%)	1 (3%)	4 (13%)	4 (13%)
		101–500	19	10 (53%)	12 (63%)	12 (63%)	7 (37%)	8 (42%)	9 (47%)
		>501	7	6 (86%)	6 (86%)	6 (86%)	6 (86%)	7 (100%)	7 (100%)

Design	—				*p* = 0.616	*p* = 0.329	*p* = 0.774	*p* = 0.427	*p* = 0.557
		Cross-Sectional	11	7 (64%)	5 (45%)	8 (73%)	3 (27%)	5 (45%)	5 (45%)
		Cohort	36	17 (47%)	21 (58%)	17 (47%)	8 (22%)	10 (28%)	11 (31%)
		Case-Studies	9	5 (56%)	6 (67%)	5 (56%)	3 (33%)	4 (44%)	4 (44%)

Journal impact factor	3.4 (1.8; 5.8)			*p* = 0.327	**p** < 0.001	*p* = 0.015	*p* = 0.111	*p* = 0.05	*p* = 0.034
		<3	27	12 (44%)	8 (30%)	10 (37%)	4 (15%)	5 (18%)	5 (18%)
		3–6	16	8 (50%)	13 (81%)	13 (81%)	4 (35%)	7 (44%)	8 (50%)
		>6.01	13	9 (69%)	11 (85%)	7 (54%)	6 (46%)	7 (54%)	7 (54%)

Yearly Scopus citations	8 (3; 16)			*p* = 0.538	*p* = 0.635	*p* = 0.203	*p* = 0.918	*p* = 0.161	*p* = 0.283
		<7	24	11 (46%)	12 (50%)	10 (42%)	6 (25%)	5 (21%)	6 (25%)
		7–16	18	9 (50%)	11 (61%)	10 (56%)	5 (28%)	7 (39%)	7 (39%)
		>16.1	14	9 (64%)	9 (64%)	10 (54%)	3 (21%)	7 (50%)	7 (50%)

## Abbreviations

ACS: Acute coronary syndrome
AMI: Acute myocardial infarction
CAD: Coronary artery disease
CV: Cardiovascular
DGCR8: Di George syndrome critical region 8
HDL: High density lipoprotein
hs-cTNT: High-sensitivity cardiac troponin T
LNA: Locked nucleic acid
miRNA: MicroRNA
mRNA: Messenger RNA
qRT-PCR: Quantitative real-time polymerase chain reaction
TRBP: TAR RNA-binding protein
VLDL: Very low density lipoprotein.
