# Supplementary material for: MicroRNAs in Coronary Heart Disease: Ready to Enter the Clinical Arena?
Source: Biomed Res Int. 2016 Nov 2;2016:2150763. doi: 10.1155/2016/2150763 (PMC5110879; doi:10.1155/2016/2150763)
Supplement: Supplementary file 1 — In the supplementary material there is the search strategy and selection criteria of the articles included in the quality reporting, Abstraction, Quality Appraisal, Effect Estimate, and Citation Count and statistical analysis methodology. Figure S1 is a schematic drawing of the selection of the included studies, based on PRISMA guidelines. In Table S1there are the full references of the studies included in the quality reporting analysis. [file 2150763.f1.docx]

**Supplement to: microRNAs in coronary heart disease: ready to enter the clinical arena?**

Elena Cavarretta^1^, Giacomo Frati^1,2^

Methods

Search and Selection

We searched for original articles on circulating microRNAs as biomarkers in coronary heart disease based on an explicit query in MEDLINE/Pubmed, last updated on July 12, 2016, performed with the Clinical Queries tool using the following as string: ("Coronary Artery Disease"[Mesh] OR "Atherosclerosis"[Mesh] OR "Myocardial Infarction"[Mesh] OR "Acute Coronary Syndrome"[Mesh]) AND ("MicroRNAs"[Mesh]) AND (Humans[Mesh] AND English[lang]). After screening at the title and abstract level, the full text of potentially eligible articles was analyzed. Reference lists of included studies were also screened for additional suitable articles. In addition, we excluded studies published in languages other than English. Figure S1 resumes the study selection.

Abstraction, Quality Appraisal, Effect Estimate, and Citation Count

All reviewing activities were performed by two independent and experienced reviewers, with divergences resolved after consensus. We extracted a comprehensive set of key study, patient, methods, main outcome, and methodology features from the included studies. We explicitly appraised article quality with the #5 items list proposed by Real [Real J, Forné C, Roso-Llorach A, Martínez-Sánchez JM. Quality Reporting of Multivariable Regression Models in Observational Studies: Review of a Representative Sample of Articles Published in Biomedical Journals. Medicine (Baltimore). 2016 May;95(20):e3653]. Finally, we obtained citation counts and computed yearly citations for each shortlisted article from Scopus. Specifically, yearly citations were computed as total citations divided by the time elapsed between publication and citational database analysis. Citation counts were last updated on July 12, 2016.

Statistical Analysis

Continuous variables are reported as median (1st quartile; 3rd quartile). Categorical variables are reported as count (percentage). For each item specified for review, prevalence estimates and 95% confidence interval (CI) using normal approximation were obtained. All analyses were stratified in groups according to the impact factor of the journal in the year of publication (2010-11; 2012-13; 2014-15; 2016), sample size (<100, 101–500, >501), design (cross-sectional, cohort, and case–control), journal impact factor (<3, 3.0-6.0, >6.01), yearly Scopus citations (<7, 7-16, >16.1). Pearson chi-square was used to assess the association between prevalence of the items of interest and the categorical secondary variables. To assess and control for possible interactions, the analysis was again stratified by impact factor, sample size, yearly citations. Significance level was set at a.0.05. All analyses were carried out using the SPSS statistical software, version 20 (IBM corporation).

Ethical statement: None required the approval of the Ethics Committee because the primary source was secondary data from published scientific articles.

Figure S1. Selection of the included studies
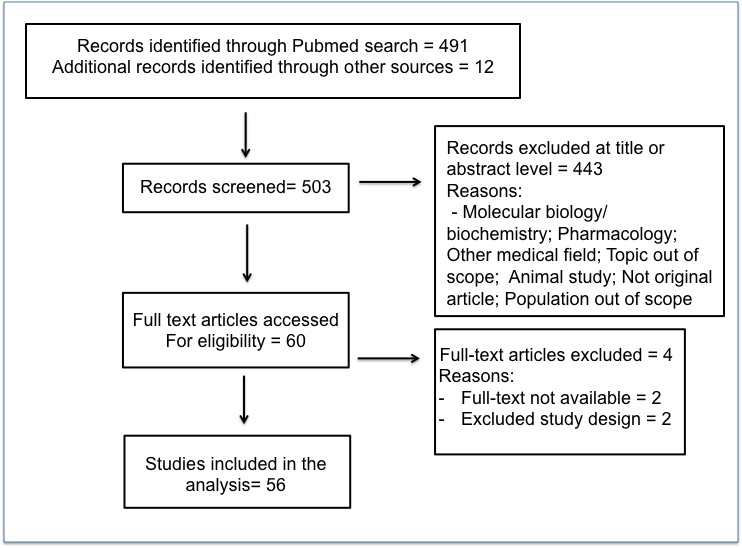


Table 1S. Full-reference studies included in the analysis.

| Reference | Year |
| --- | --- |
| Ai J, Zhang R, Li Y, Pu J, Lu Y, Jiao J, Li K, Yu B, Li Z, Wang R, Wang L, Li Q, Wang N, Shan H, Li Z, Yang B. Circulating microRNA-1 as a potential novel biomarker for acute myocardial infarction. Biochem Biophys Res Commun. 2010;391(1):73-7. | 2010 |
| D'Alessandra Y, Devanna P, Limana F, Straino S, Di Carlo A, Brambilla PG, Rubino M, Carena MC, Spazzafumo L, De Simone M, Micheli B, Biglioli P, Achilli F, Martelli F, Maggiolini S, Marenzi G, Pompilio G, Capogrossi MC. Circulating microRNAs are new and sensitive biomarkers of myocardial infarction. Eur Heart J. 2010;31(22):2765-73. | 2010 |
| Wang GK, Zhu JQ, Zhang JT, Li Q, Li Y, He J, Qin YW, Jing Q. Circulating microRNA: a novel potential biomarker for early diagnosis of acute myocardial infarction in humans. Eur Heart J. 2010;31(6):659-66. | 2010 |
| Corsten MF, Dennert R, Jochems S, Kuznetsova T, Devaux Y, Hofstra L, Wagner DR, Staessen JA, Heymans S, Schroen B. Circulating MicroRNA-208b and MicroRNA-499 reflect myocardial damage in cardiovascular disease. Circ Cardiovasc Genet. 2010;3(6):499-506. | 2010 |
| Adachi T, Nakanishi M, Otsuka Y, Nishimura K, Hirokawa G, Goto Y, Nonogi H, Iwai N. Plasma microRNA 499 as a biomarker of acute myocardial infarction. Clin Chem. 2010 Jul;56(7):1183-5. | 2010 |
| Fichtlscherer S, De Rosa S, Fox H, Schwietz T, Fischer A, Liebetrau C, Weber M, Hamm CW, Röxe T, Müller-Ardogan M, Bonauer A, Zeiher AM, Dimmeler S. Circulating microRNAs in patients with coronary artery disease. Circ Res. 2010;107(5):677-84. | 2010 |
| Hoekstra M, van der Lans CA, Halvorsen B, Gullestad L, Kuiper J, Aukrust P, van Berkel TJ, Biessen EA. The peripheral blood mononuclear cell microRNA signature of coronary artery disease. Biochem Biophys Res Commun. 2010 Apr 9;394(3):792-7. | 2010 |
| Kuwabara Y, Ono K, Horie T, Nishi H, Nagao K, Kinoshita M, Watanabe S, Baba O, Kojima Y, Shizuta S, Imai M, Tamura T, Kita T, Kimura T. Increased microRNA-1 and microRNA-133a levels in serum of patients with cardiovascular disease indicate myocardial damage. Circ Cardiovasc Genet. 2011;4(4):446-54. | 2011 |
| Wang R, Li N, Zhang Y, Ran Y, Pu J. Circulating microRNAs are promising novel biomarkers of acute myocardial infarction. Intern Med 2011;50:1789–95. | 2011 |
| Meder B, Keller A, Vogel B, et al.MicroRNA signatures in total peripheral blood as novel biomarkers for acute myocardial infarction. Basic Res Cardiol 2011;106:13–23. | 2011 |
| De Rosa S, Fichtlscherer S, Lehmann R, Assmus B, Dimmeler S, Zeiher AM. Transcoronary concentration gradients of circulating microRNAs. Circulation. 2011 Nov 1;124(18):1936-44. | 2011 |
| Widera C, Gupta SK, Lorenzen JM, Bang C, Bauersachs J, Bethmann K, Kempf T, Wollert KC, Thum T. Diagnostic and prognostic impact of six circulating microRNAs in acute coronary syndrome. J Mol Cell Cardiol. 2011 Nov;51(5):872-5. | 2011 |
| Weber M, Baker MB, Patel RS, Quyyumi AA, Bao G, Searles CD. MicroRNA Expression Profile in CAD Patients and the Impact of ACEI/ARB. Cardiol Res Pract 2011;2011:1–5. | 2011 |
| Long G, Wang F, Duan Q, et al. Human circulating microRNA-1 and microRNA-126 as potential novel indicators for acute myocardial infarction. Int J Biol Sci 2012;8:811–8. | 2012 |
| Devaux Y, Vausort M, Goretti E, et al. Use of circulating microRNAs to diagnose acute myocardial infarction. Clin Chem 2012;58:559–67. | 2012 |
| Long G, Wang F, Duan Q, Yang S, Chen F, Gong W, Yang X, Wang Y, Chen C, Wang DW. Circulating miR-30a, miR-195 and let-7b associated with acute myocardial infarction. PLoS One. 2012;7(12):e50926. | 2012 |
| Eitel I, Adams V, Dieterich P, Fuernau G, de Waha S, Desch S, Schuler G, Thiele H. Relation of circulating MicroRNA-133a concentrations with myocardial damage and clinical prognosis in ST-elevation myocardial infarction. Am Heart J. 2012 Nov;164(5):706-14. | 2012 |
| Olivieri F, Antonicelli R, Lorenzi M, et al. Diagnostic potential of circulating miR-499-5p in elderly patients with acute non ST-elevation myocardial infarction. Int J Cardiol. 2013 Jul 31;167(2):531-6. | 2012 |
| Zampetaki A, Willeit P, Tilling L, Drozdov I, Prokopi M, Renard JM, Mayr A, Weger S, Schett G, Shah A, Boulanger CM, Willeit J, Chowienczyk PJ, Kiechl S, Mayr M. Prospective study on circulating MicroRNAs and risk of myocardial infarction. J Am Coll Cardiol. 2012 Jul 24;60(4):290-9. | 2012 |
| Gao W, He HW, Wang ZM, Zhao H, Lian XQ, Wang YS, Zhu J, Yan JJ, Zhang DG, Yang ZJ, Wang LS. Plasma levels of lipometabolism-related miR-122 and miR-370 are increased in patients with hyperlipidemia and associated with coronary artery disease. Lipids Health Dis. 2012;11:55. | 2012 |
| Matsumoto S, Sakata Y, Nakatani D, Suna S, Mizuno H, Shimizu M, Usami M, Sasaki T, Sato H, Kawahara Y, Hamasaki T, Nanto S, Hori M, Komuro I. A subset of circulating microRNAs are predictive for cardiac death after discharge for acute myocardial infarction. Biochem Biophys Res Commun. 2012;427(2):280-4. | 2012 |
| Oerlemans MI, Mosterd A, Dekker MS, de Vrey EA, van Mil A, Pasterkamp G, Doevendans PA, Hoes AW, Sluijter JP. Early assessment of acute coronary syndromes in the emergency department: the potential diagnostic value of circulating microRNAs. EMBO Mol Med. 2012;4(11):1176-85. | 2012 |
| Sun X, Zhang M, Sanagawa A, Mori C, Ito S, Iwaki S, Satoh H, Fujii S. Circulating microRNA-126 in patients with coronary artery disease: correlation with LDL cholesterol. Thromb J. 2012;10(1):16. | 2012 |
| Liebetrau C, Möllmann H, Dörr O, Szardien S, Troidl C, Willmer M, Voss S, Gaede L, Rixe J, Rolf A, Hamm C, Nef H. Release kinetics of circulating muscle-enriched microRNAs in patients undergoing transcoronary ablation of septal hypertrophy. J Am Coll Cardiol. 2013;62(11):992-8. | 2013 |
| Goretti E, Vausort M, Wagner DR, Devaux Y. Association between circulating microRNAs, cardiovascular risk factors and outcome in patients with acute myocardial infarction. Int J Cardiol. 2013;168(4):4548-50. | 2013 |
| Wang F, Long G, Zhao C, Li H, Chaugai S, Wang Y, Chen C, Wang DW. Plasma microRNA-133a is a new marker for both acute myocardial infarction and underlying coronary artery stenosis. J Transl Med. 2013 Sep 23;11:222. | 2013 |
| Gidlöf O, Smith JG, Miyazu K, Gilje P, Spencer A, Blomquist S, Erlinge D. Circulating cardio-enriched microRNAs are associated with long-term prognosis following myocardial infarction. BMC Cardiovasc Disord. 2013 Feb 28;13:12. | 2013 |
| Li C, Fang Z, Jiang T, Zhang Q, Liu C, Zhang C, Xiang Y. Serum microRNAs profile from genome-wide serves as a fingerprint for diagnosis of acute myocardial infarction and angina pectoris. BMC Med Genomics. 2013 May 4;6:16. | 2013 |
| Lu HQ, Liang C, He ZQ, Fan M, Wu ZG. Circulating miR-214 is associated with the severity of coronary artery disease. J Geriatr Cardiol. 2013 Mar;10(1):34-8. | 2013 |
| D'Alessandra Y, Carena MC, Spazzafumo L, Martinelli F, Bassetti B, Devanna P, Rubino M, Marenzi G, Colombo GI, Achilli F, Maggiolini S, Capogrossi MC, Pompilio G. Diagnostic potential of plasmatic MicroRNA signatures in stable and unstable angina. PLoS One. 2013 Nov 15;8(11):e80345. | 2013 |
| Finn NA, Eapen D, Manocha P, Al Kassem H, Lassegue B, Ghasemzadeh N, et al. Coronary heart disease alters intercellular communication by modifying microparticle-mediated microRNA transport. FEBS Lett. 2013;587:3456–63 | 2013 |
| Jaguszewski M, Osipova J, Ghadri JR, Napp LC, Widera C, Franke J, Fijalkowski M, Nowak R, Fijalkowska M, Volkmann I, Katus HA, Wollert KC, Bauersachs J, Erne P, Lüscher TF, Thum T, Templin C. A signature of circulating microRNAs differentiates takotsubo cardiomyopathy from acute myocardial infarction. Eur Heart J. 2014;35(15):999-1006. | 2014 |
| Zeller T, Keller T, Ojeda F, Reichlin T, Twerenbold R, Tzikas S, Wild PS, Reiter M, Czyz E, Lackner KJ, Munzel T, Mueller C, Blankenberg S. Assessment of microRNAs in patients with unstable angina pectoris. Eur Heart J. 2014;35(31):2106-14. | 2014 |
| He F, Lv P, Zhao X, Wang X, Ma X, Meng W, Meng X, Dong S. Predictive value of circulating miR-328 and miR-134 for acute myocardial infarction. Mol Cell Biochem. 2014;394(1-2):137-44. | 2014 |
| Hsu A, Chen SJ, Chang YS, Chen HC, Chu PH. Systemic approach to identify serum microRNAs as potential biomarkers for acute myocardial infarction. Biomed Res Int. 2014;2014:418628. | 2014 |
| Zhu GF, Yang LX, Guo RW, Liu H, Shi YK, Ye JS, Yang ZH. microRNA-155 is inversely associated with severity of coronary stenotic lesions calculated by the Gensini score. Coron Artery Dis. 2014 Jun;25(4):304-10. | 2014 |
| Jansen F, Yang X, Proebsting S, Hoelscher M, Przybilla D, Baumann K, Schmitz T, Dolf A, Endl E, Franklin BS, Sinning JM, Vasa-Nicotera M, Nickenig G, Werner N. MicroRNA expression in circulating microvesicles predicts cardiovascular events in patients with coronary artery disease. J Am Heart Assoc. 2014 Oct 27;3(6):e001249. | 2014 |
| Jiang Y, Wang HY, Li Y, Guo SH, Zhang L, Cai JH. Peripheral blood miRNAs as a biomarker for chronic cardiovascular diseases. Sci Rep. 2014 May 22;4:5026. | 2014 |
| Pilbrow AP, Cordeddu L, Cameron VA, Frampton CM, Troughton RW, Doughty RN, Whalley GA, Ellis CJ, Yandle TG, Richards AM, Foo RS-Y. Circulating miR-323-3p and miR-652: candidate markers for the presence and progression of acute coronary syndromes. Int J Cardiol 2014;176:375–385. | 2014 |
| Wang F, Long G, Zhao C, Li H, Chaugai S, Wang Y, Chen C, Wang DW. Atherosclerosis-related circulating miRNAs as novel and sensitive predictors for acute myocardial infarction. PloS One 2014;9:e105734 | 2014 |
| Huang S, Chen M, Li L, He M, Hu D, Zhang X, Li J, Tanguay RM, Feng J, Cheng L, Zeng H, Dai X, Deng Q, Hu FB, Wu T. Circulating MicroRNAs and the occurrence of acute myocardial infarction in Chinese populations. Circ Cardiovasc Genet. 2014 Apr;7(2):189-98. | 2014 |
| Li L-M, Cai W-B, Ye Q, Liu J-M, Li X, Liao X-X. Comparison of plasma microRNA-1 and cardiac troponin T in early diagnosis of patients with acute myocardial infarction. World J Emerg Med 2014;5:182–186. | 2014 |
| Li Z, Lu J, Luo Y, Li S, Chen M. High association between human circulating microRNA-497 and acute myocardial infarction. ScientificWorldJournal. 2014;2014:931845. | 2014 |
| Liu F, Li R, Zhang Y, Qiu J, Ling W. Association of plasma MiR-17-92 with dyslipidemia in patients with coronary artery disease. Medicine (Baltimore). 2014 Nov;93(23):e98. | 2014 |
| Wang J, Pei Y, Zhong Y, Jiang S, Shao J, Gong J. Altered serum microRNAs as novel diagnostic biomarkers for atypical coronary artery disease. PLoS One. 2014 Sep 8;9(9):e107012. | 2014 |
| Zhong J, He Y, Chen W, Shui X, Chen C, Lei W. Circulating microRNA-19a as a potential novel biomarker for diagnosis of acute myocardial infarction. Int J Mol Sci. 2014 Nov 6;15(11):20355-64. | 2014 |
| Devaux Y, Mueller M, Haaf P, Goretti E, Twerenbold R, Zangrando J, Vausort M, Reichlin T, Wildi K, Moehring B, Wagner DR, Mueller C. Diagnostic and prognostic value of circulating microRNAs in patients with acute chest pain. J Intern Med. 2015;277(2):260-71. | 2015 |
| Dong YM, Liu XX, Wei GQ, Da YN, Cha L, Ma CS. Prediction of long-term outcome after acute myocardial infarction using circulating miR-145. Scand J Clin Lab Invest. 2015;75(1):85-91. | 2015 |
| Gao H, Guddeti RR, Matsuzawa Y, Liu LP, Su LX, Guo D, Nie SP, Du J, Zhang M. Plasma Levels of microRNA-145 Are Associated with Severity of Coronary Artery Disease. PLoS One. 2015 May 4;10(5):e0123477. | 2015 |
| Zhang L, Chen X, Su T, Li H, Huang Q, Wu D, Yang C, Han Z. Circulating miR-499 are novel and sensitive biomarker of acute myocardial infarction. J Thorac Dis. 2015 Mar;7(3):303-8. | 2015 |
| Zhang R, Lan C, Pei H, Duan G, Huang L, Li L. Expression of circulating miR-486 and miR-150 in patients with acute myocardial infarction. BMC Cardiovasc Disord. 2015 Jun 16;15:51. | 2015 |
| Jin Y, Yang CJ, Xu X, Cao JN, Feng QT, Yang J. MiR-214 regulates the pathogenesis of patients with coronary artery disease by targeting VEGF. Mol Cell Biochem. 2015 Apr;402(1-2):111-22. | 2015 |
| Li C, Chen X, Huang J, Sun Q, Wang L. Clinical impact of circulating miR-26a, miR-191, and miR-208b in plasma of patients with acute myocardial infarction. Eur J Med Res. 2015 Jun 5;20:58. | 2015 |
| Liu J, Liu Y, Sun YN, Li S, Liu XQ, Li J, Li CM, Tian W, Zhou YT, Shang XM. miR-28-5p Involved in LXR-ABCA1 Pathway is Increased in the Plasma of Unstable Angina Patients. Heart Lung Circ. 2015 Jul;24(7):724-30. | 2015 |
| Yao XL, Lu XL, Yan CY, Wan QL, Cheng GC, Li YM. Circulating miR-122-5p as a potential novel biomarker for diagnosis of acute myocardial infarction. Int J Clin Exp Pathol. 2015 Dec 1;8(12):16014-9. | 2015 |
| Zhao CH, Cheng GC, He RL, Hong Y, Wan QL, Wang ZZ, Pan ZY. Analysis and clinical significance of microRNA-499 expression levels in serum of patients with acute myocardial infarction. Genet Mol Res. 2015 Apr 27;14(2):4027-34. | 2015 |
| Karakas M, Schulte C, Appelbaum S, Ojeda F, Lackner KJ, Münzel T, Schnabel RB, Blankenberg S, Zeller T. Circulating microRNAs strongly predict cardiovascular death in patients with coronary artery disease-results from the large AtheroGene study. Eur Heart J. 2016 Jun 29. pii: ehw250. [Epub ahead of print] | 2016 |
| Gacoń J, Kabłak-Ziembicka A, Stępień E, Enguita FJ, Karch I, Derlaga B, Żmudka K, Przewłocki T. Decision-making microRNAs (miR-124, -133a/b, -34a and -134) in patients with occluded target vessel in acute coronary syndrome. Kardiol Pol. 2016;74(3):280-8. | 2016 |
